# Supplementary material for: Rational inattention and tonic dopamine
Source: PLoS Comput Biol. 2021 Mar 24;17(3):e1008659. doi: 10.1371/journal.pcbi.1008659 (PMC7990190; doi:10.1371/journal.pcbi.1008659)
Supplement: S1 Appendix — (PDF) [file pcbi.1008659.s001.pdf]

Rational Inattention and Tonic Dopamine

John G. Mikhael, Lucy Lai, Samuel J. Gershman

# S1 Appendix. The scalar property as a consequence of rational inattention.

We outline here a derivation of the scalar property (Weber's law) based on rational inattention principles. The purpose of this derivation is to provide a mathematically well-specified model of timing noise (or likelihood precision). Quantitatively, this derivation will control the observed increase in timing noise for larger intervals, but our main effects (central tendency and changes in clock speed) do not depend on this derivation, and only depend on the assumption that the control of precision be implemented via changes in clock speed.

In Eq 10 in the main text, we derived the relationship between reward, information cost, and precision. When the temporal task is not highly conditioned, $\lambda_0 \simeq 0$, and we can simply write

$$\lambda \simeq \frac{2R}{\kappa}. \tag{1}$$

(In highly conditioned tasks, on the other hand, $\lambda_0$ becomes large, the central tendency effect becomes prominent, and the scalar property is not guaranteed to apply [1].)

In interval timing tasks, rewards are subject to hyperbolic discounting [2–4]:

$$R = \frac{R_0}{t+1}, \tag{2}$$

where $R_0$ is the undiscounted reward, which in our framework is reported by DA, and $t$ is time. Following previous work [5], we assume that the cost of collecting information (reducing noise) scales with the attended

duration, and we take this scaling to be linear:

$$\kappa = \kappa_0 t. \tag{3}$$

Here, $\kappa_0$ represents the attentional cost per unit time. It follows then that

$$\lambda \simeq \left(\frac{2R_0}{\kappa_0}\right)\frac{1}{t^2}. \tag{4}$$

This is the scalar property, with Weber fraction given by:

$$w \simeq \sqrt{\frac{\kappa_0}{2R_0}}. \tag{5}$$

This leaves open the question of how the scalar property is actually implemented. One approach is to take a concave (e.g., logarithmic) mapping of objective time to subjective (psychological) time:

$$\tau = \eta \log{(t+1)}, \tag{6}$$

where $\tau$ represents subjective time, and $\eta$ is a scaling factor, which can be interpreted as the pacemaker rate of the internal clock [6–8]. We hold subjective precision $l$ constant. Bayesian update occurs in subjective time, and is subsequently mapped back to objective time during decoding.

For any desired precision $\lambda$, we can then find $\eta$ to achieve this precision. In particular, by deriving the relationship between subjective and objective precision:

$$\lambda = l\left(\frac{\eta}{t+1}\right)^2, \tag{7}$$

it follows that

$$\eta \simeq \sqrt{\frac{2R_0}{l\kappa_0}}. \tag{8}$$

Hence, average reward $R_0$ controls precision $\lambda$ by setting $\eta$ (e.g., see Fig S1). In particular, the speed of the clock increases with reward ($R_0$) and decreases with cost ($\kappa_0$).

Interestingly, this framework predicts that the scalar property should not apply over all time ranges, but rather, the ratio $\frac{2R}{\kappa}$ eventually becomes small enough that $\lambda_0$ cannot be disregarded, and eventually, $\lambda = \max(0, \frac{2R}{\kappa} - \lambda_0) = 0$. After that point, it is no longer worth using this mechanism to time. Instead, a different timing mechanism in which $\kappa$ does not increase linearly should apply. This may explain why interval timing

only applies over the seconds-to-hours range [9].

A second prediction of this derivation is that changes in precision will be more evident under changes to the reward rate, rather than the reward magnitude. To see this, assume the timed duration at baseline is $T$, and the average reward is increased from $R$ to $nR > R$. If this change occurred by increasing reward *rate*, then the duration to be attended to before each reward is delivered becomes $\frac{T}{n}$, and the magnitude of each reward remains $R$. Hence for each reward, both the cost and reward discounting scale by $\frac{1}{n}$. On the other hand, if this change occurred by increasing reward *magnitude*, then the magnitude increases to $nR$, but the duration attended to remains $T$. Hence cost and reward discounting are unchanged. Let us compute the new precisions, $\lambda_r$ and $\lambda_m$, respectively:

$$\lambda_r \simeq \frac{2R}{(\kappa/n)(1/n)} = \frac{2n^2R}{\kappa}. \tag{9}$$

On the other hand,

$$\lambda_m \simeq \frac{2nR}{\kappa}. \tag{10}$$

Therefore, $\lambda_r > \lambda_m$.

In fact, subjective reward magnitudes are concave with objective magnitudes, rather than linear [10, 11]. This further amplifies the difference between the two precisions: In this case,

$$\lambda_r = \frac{2nU(R)}{\kappa/n} = \frac{2n^2U(R)}{\kappa}, \tag{11}$$

where $U(R)$ represents the subjective value of reward $R$ (the utility of $R$), which is a concave function. On the other hand,

$$\lambda_m = \frac{2U(nR)}{\kappa}. \tag{12}$$

This result predicts that $\lambda_r >> \lambda_m$. Indeed, changes in reward rate have much more profound effects on interval timing than changes in reward magnitude [12, 13].

# References


1. Jazayeri M, Shadlen MN. Temporal context calibrates interval timing. Nature neuroscience. 2010;13(8):1020.

2. Frederick S, Loewenstein G, O'donoghue T. Time discounting and time preference: A critical review. Journal of economic literature. 2002;40(2):351–401.

3. Sozou PD. On hyperbolic discounting and uncertain hazard rates. Proceedings of the Royal Society of London Series B: Biological Sciences. 1998;265(1409):2015–2020.

4. Madden GJ, Begotka AM, Raiff BR, Kastern LL. Delay discounting of real and hypothetical rewards. Experimental and clinical psychopharmacology. 2003;11(2):139.

5. Gershman SJ, Bhui R. Rationally inattentive intertemporal choice. Nature communications. 2020;11(1):1–8.

6. Gibbon J, Malapani C, Dale CL, Gallistel C. Toward a neurobiology of temporal cognition: advances and challenges. Current opinion in neurobiology. 1997;7(2):170–184.

7. Treisman M. Temporal discrimination and the indifference interval: Implications for a model of the "internal clock". Psychological Monographs: General and Applied. 1963;77(13):1.

8. Zakay D, Block RA. Temporal cognition. Current directions in psychological science. 1997;6(1):12–16.

9. Buhusi CV, Meck WH. What makes us tick? Functional and neural mechanisms of interval timing. Nature Reviews Neuroscience. 2005;6(10):755–765.

10. Bernoulli D. Exposition of a new theory on the measurement of risk. Econometrica. 1954;22(1):23–36.

11. Kahneman D, Tversky A. Prospect theory: An analysis of decision under risk. Econometrica: Journal of the Econometric Society. 1979; p. 263–291.

12. Killeen PR, Fetterman JG. A behavioral theory of timing. Psychological review. 1988;95(2):274.

13. Galtress T, Kirkpatrick K. Reward value effects on timing in the peak procedure. Learning and Motivation. 2009;40(2):109–131.
