# Supplementary material for: Rational inattention and tonic dopamine
Source: PLoS Comput Biol. 2021 Mar 24;17(3):e1008659. doi: 10.1371/journal.pcbi.1008659 (PMC7990190; doi:10.1371/journal.pcbi.1008659)
Supplement: S2 Appendix — (PDF) [file pcbi.1008659.s002.pdf]

Rational Inattention and Tonic Dopamine

John G. Mikhael, Lucy Lai, Samuel J. Gershman

S2 Appendix. Time cell rescaling.

In the previous section, we showed that average reward R_0, which in our model is reported by DA, controls precision λ by setting the pacemaker rate η. As shown in Fig S1, the temporal receptive field surrounding any given objective time tightens under higher DA levels.

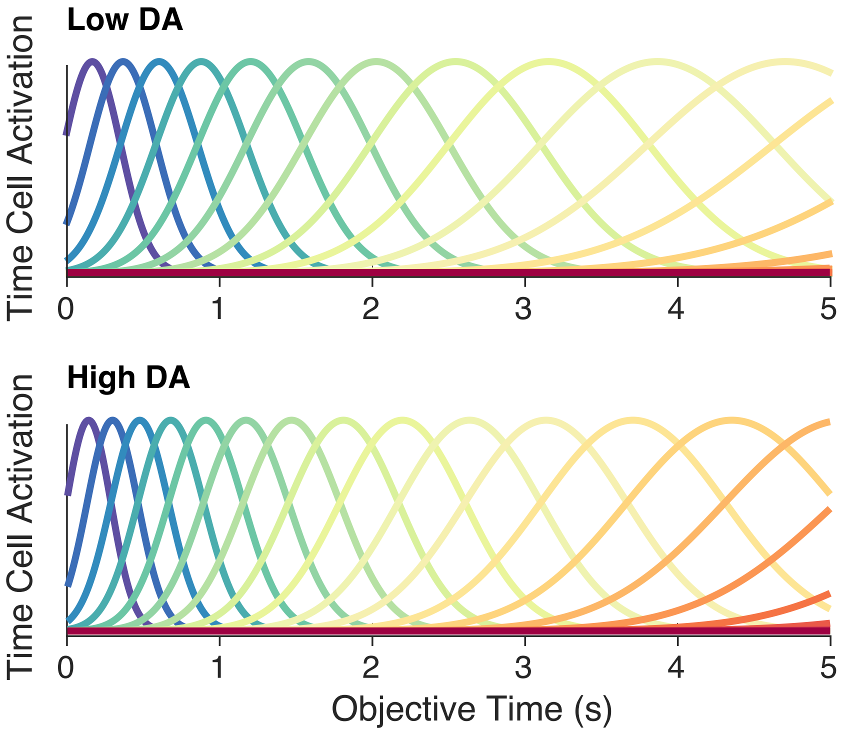

Fig S1. Time cell receptive fields rescale to increase precision. Mathematically, manipulations of precision correspond to changing the mapping between subjective and objective time. Simulation details: We have chosen $\kappa_0 = 0.05s^{-1}$, $\lambda = 1$ in subjective time, and DA = 1 and 1.5 for the low and high DA conditions, respectively.
