# Supplementary material for: Rational inattention and tonic dopamine
Source: PLoS Comput Biol. 2021 Mar 24;17(3):e1008659. doi: 10.1371/journal.pcbi.1008659 (PMC7990190; doi:10.1371/journal.pcbi.1008659)
Supplement: S3 Appendix — (PDF) [file pcbi.1008659.s003.pdf]

Rational Inattention and Tonic Dopamine

John G. Mikhael, Lucy Lai, Samuel J. Gershman

S3 Appendix. Interval timing in discrimination tasks.

Interval timing is typically studied using production tasks [1], such as the experiments considered in the main text, or discrimination tasks [2]. In discrimination tasks, subjects (or animals) learn to respond differently to short-duration stimuli and long-duration stimuli. Intermediate-duration stimuli are then presented during probe trials, and for each, the subjects must respond with the short-duration response or the long-duration response. Subsequent changes in their response profiles following certain manipulations (e.g., administration of drugs) allow us to infer changes in their internal timing system.

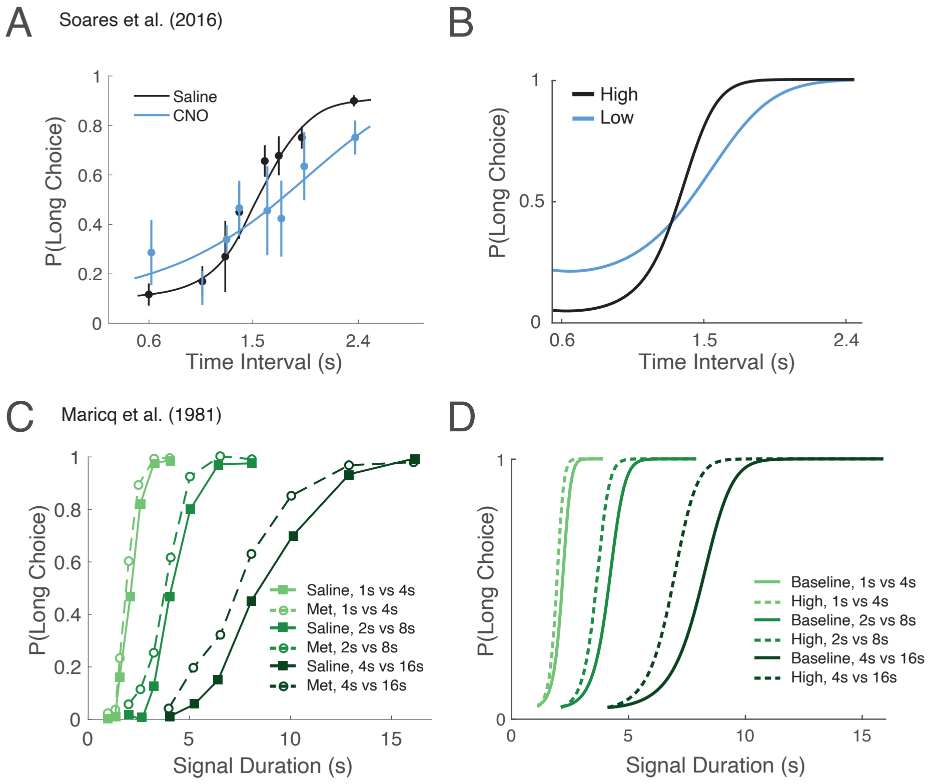

Fig S2. Rational inattention and interval timing in discrimination tasks. (A) Soares et al. [3] trained mice on a temporal discrimination task in which they reported intervals of variable duration as either shorter or longer than 1.5 seconds. When DA activity was pharmacogenetically suppressed, the discrimination curve flattened. p(Long Choice): probability of judging a duration as long; CNO: clozapine N-oxide. Figure adapted from [3]. (B) Our model recapitulates this effect: Under rational inattention, high DA increases precision, which mitigates Bayesian migration and improves discrimination. Note here, however, that motivation is a confounding factor. (C) Maricq et al. [4] have shown that, in rats, acute administration of methamphetamine (DA agonist) during testing in discrimination tasks with various time pairs biased estimation toward the 'long' response. Pairs of solid and dashed curves represent conditions where the short and long durations were 1 and 4 seconds (light green), 2 and 8 seconds (green), and 4 and 16 seconds (dark green). This discrimination paradigm controls for motivation, a potential confound in reproduction tasks (Fig 5C in the main text). Figure adapted from [4]. (D) Our model recapitulates this effect: High DA at decoding increases the speed of the clock, which biases estimation of duration toward longer responses. Simulation details: We have chosen $\kappa_0 = 0.2s^{-1}$, and DA levels of 0.3, 0.7 and 0.8 for low, baseline, and high conditions, respectively. Average reward was set to the DA level. Parameter tuning: For both experiments, the qualitative results hold for any choice of $\frac{R}{\kappa} > 0$, $l > 0$, $\lambda_0 > 0$, and DA > 0, such that $\frac{2R}{\kappa} > \lambda_0$ (after Eq 10 in the main text).

References

1. Roberts S. Isolation of an internal clock. Journal of Experimental Psychology: Animal Behavior Processes. 1981;7(3):242.

2. Church RM, Deluty MZ. Bisection of temporal intervals. Journal of Experimental Psychology: Animal Behavior Processes. 1977;3(3):216.

3. Soares S, Atallah BV, Paton JJ. Midbrain dopamine neurons control judgment of time. Science. 2016;354(6317):1273–1277.

4. Maricq AV, Roberts S, Church RM. Methamphetamine and time estimation. Journal of Experimental Psychology: Animal Behavior Processes. 1981;7(1):18.
