# Supplementary material for: Rational inattention and tonic dopamine
Source: PLoS Comput Biol. 2021 Mar 24;17(3):e1008659. doi: 10.1371/journal.pcbi.1008659 (PMC7990190; doi:10.1371/journal.pcbi.1008659)
Supplement: S4 Appendix — (PDF) [file pcbi.1008659.s004.pdf]

Rational Inattention and Tonic Dopamine

John G. Mikhael, Lucy Lai, Samuel J. Gershman

S4 Appendix. Interval timing under average reward manipulations.

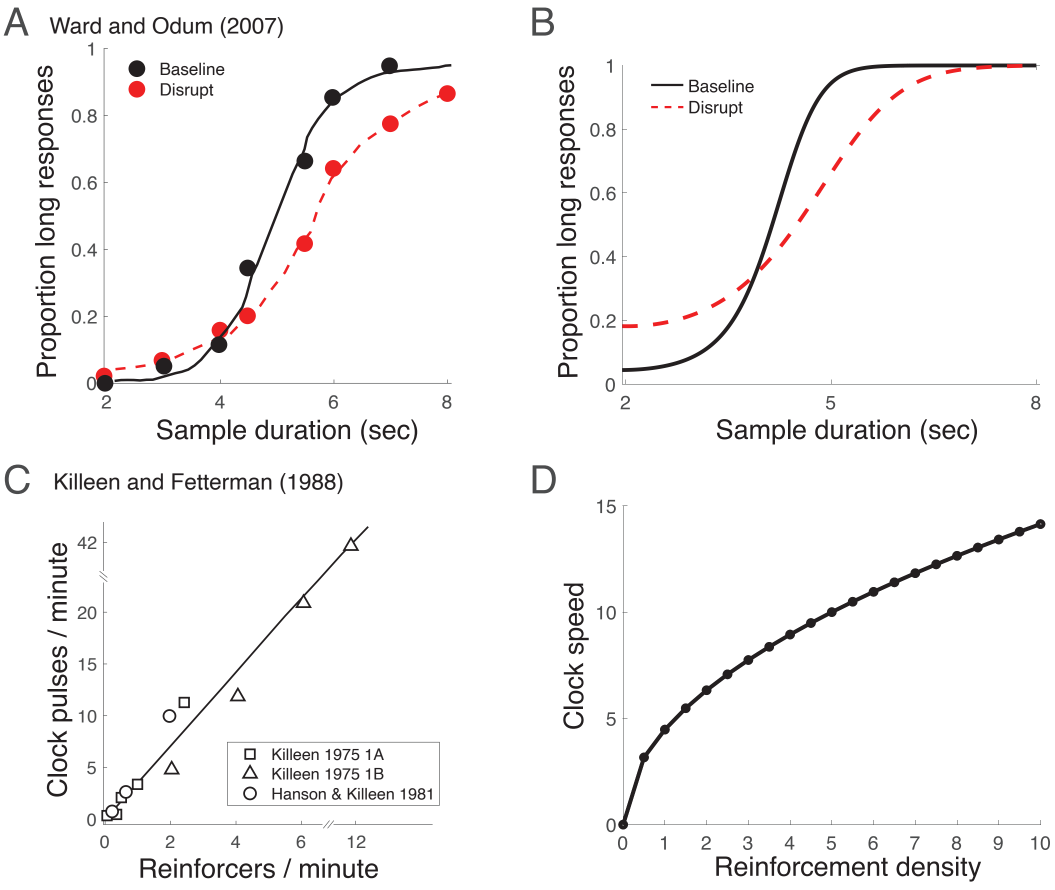

Fig S3. Effects of average reward and satiety on interval timing. (A) Ward and Odum [1] trained pigeons on a temporal discrimination task in which they reported intervals of variable duration as either shorter or longer than 5 seconds. When the animals were prefed, the discrimination curve flattened. Figure adapted from [1]. (B) Our model recapitulates this effect: Under rational inattention, high reward incentives increase precision, which mitigate Bayesian migration and improve discrimination. Note here, however, that motivation is a confounding factor. (C) Killeen and Fetterman [2] analyzed data from three experiments in which reinforcer rates were varied, and timing behavior was measured. The authors fit behavior to a model in which the speed of the internal clock could be manipulated. They showed that the clock speed increased as the reinforcer rate increased. Clock pulses/minute: Model parameter representing clock speed. Figure adapted from [2]. (D) Our model recapitulates this effect: Under rational inattention, high average reward increases precision. This results in a faster internal clock. Simulation details: We have chosen $\kappa_0 = 0.2s^{-1}$, and DA levels of 0.3 and 0.7 for high and low satiety, respectively. Average reward was set to the DA level. Parameter tuning: For both experiments, the qualitative results hold for any choice of $\frac{R}{\kappa} > 0$, $l > 0$, $\lambda_0 > 0$, and DA > 0, such that $\frac{2R}{\kappa} > \lambda_0$ (after Eq 10 in the main text).

References

1. Ward RD, Odum AL. Disruption of temporal discrimination and the choose-short effect. Animal Learning & Behavior. 2007;35(1):60–70.

2. Killeen PR, Fetterman JG. A behavioral theory of timing. Psychological review. 1988;95(2):274.
