# Supplementary material for: Rational inattention and tonic dopamine
Source: PLoS Comput Biol. 2021 Mar 24;17(3):e1008659. doi: 10.1371/journal.pcbi.1008659 (PMC7990190; doi:10.1371/journal.pcbi.1008659)
Supplement: S5 Appendix — (PDF) [file pcbi.1008659.s005.pdf]

Rational Inattention and Tonic Dopamine

John G. Mikhael, Lucy Lai, Samuel J. Gershman

S5 Appendix. Inverse U-shaped relationship between DA and performance.

We assume here that true precision λ cannot increase arbitrarily, and instead is bounded by $\lambda \leq L$, for some constant L. We will show that if the estimated precision λ' can increase beyond L, then performance will follow an inverse U-shaped curve with DA level. Define d as the desired (or targeted) precision, controlled by DA. Then $\lambda = \min(d, L)$, and $\lambda' = d$.

Combining Eqs 11 and 12 in the main text, and knowing that $c = \lambda'/\lambda$, we can rewrite the marginal error as a function of d, which is monotonic with DA:

$$E(d) = \frac{\lambda_0}{(\lambda_0 + d)^2} + \frac{d^2}{(\lambda_0 + d)^2 \min(L, d)}. \tag{1}$$

When $d \leq L$, $E(d) = \frac{1}{\lambda_0 + d}$. Hence, over this domain, error will decrease as DA increases. However, when DA increases enough for d to surpass L,

$$E(d) = \frac{\lambda_0}{(\lambda_0 + d)^2} + \frac{d^2}{(\lambda_0 + d)^2 L}. \tag{2}$$

Taking the partial derivative of E with respect to d,

$$\frac{\partial E}{\partial d} = \frac{2\lambda_0}{(\lambda_0 + d)^3} \left(\frac{d}{L} - 1 \right), \tag{3}$$

which is positive when $d > L$. Hence, error decreases with DA until true precision reaches the limit L. After this point, increases in DA increase error (Fig S4). In other words, the relationship between DA and performance takes the shape of an inverted U.

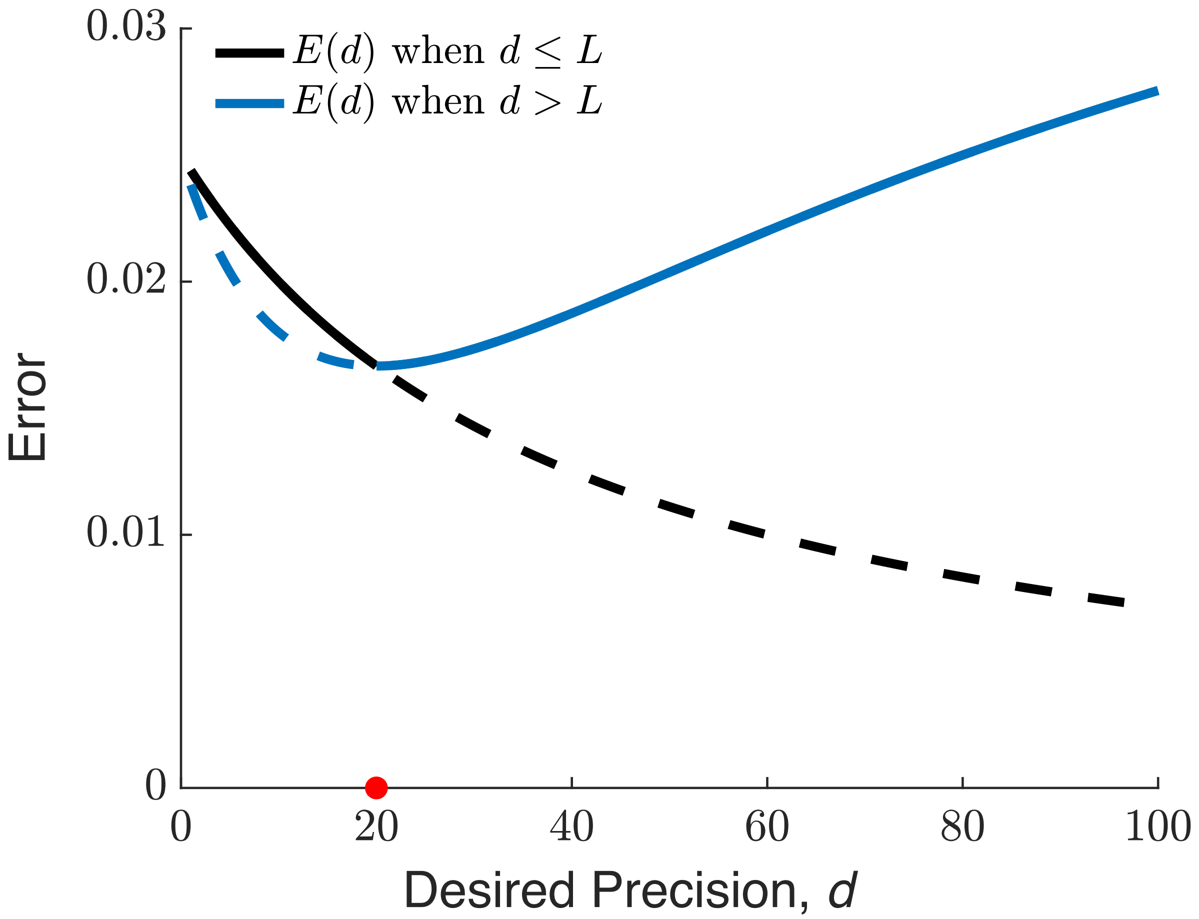

Fig S4. When true precision is bounded from above, the relationship between DA and error is U-shaped. When DA is low, increases to DA will improve both true and estimated precision, which improves performance. However, when DA increases beyond the capacity of true precision to increase, precision miscalibration will cause performance to worsen (see 'Precision miscalibration' section in the main text). Because performance depends inversely on error, it follows that the relationship between DA and precision is inverse U-shaped. Dashed curves denote the error if precision were not bounded from above (black) and if true precision were fixed at L independent of the desired precision (blue). For illustration, we have chosen $L = 20$ and $\lambda_0 = 40$. d: desired precision set by DA; $E(d)$: error as a function of desired precision; L: limit to true precision, indicated by red dot.
